# Average Occupied Beds at St. George's Hospital

**Published:** 1919-08-16

**Authors:** Jas. M. Churchfield

**Affiliations:** Secretary-Superintendent. St. George's Hospital, S.W. 1.


THE EDITOR'S LETTER-BOX—*(continued)*.

### Average Occupied Beds at St. George's Hospital.

*To the Editor of* The Hospital.

Sir,—Would you kindly note that there is an error in the criticism of this Hospital's Annual Report for 1918 on p. 462 of this week's Hospital? The daily average number of occupied beds is stated to have been 284, whereas it was 299.82, practically 300, as will be seen on reference to p. 8 and to the statistical tables on p. 43 of the Annual Report. Even this corrected figure is low compared with other years, and I would again refer you to the Annual Report, pp. 11 and 12, for the explanation. I think you will agree that in fairness to the institution this should have been published in place of the mere assumption given in your paragraph.—Yours faithfully,

(Signed) Jas. M. Churchfield,

Secretary-Superintendent.

St. George's Hospital, S.W. 1. August 2, 1919.

[We regret we incorrectly took the figure representing the total of patients in the hospital on December 31 (284) for the average bed occupation for the year. The latter figure was actually 299.82. The average was low in 1918, but more wards than usual were closed for annual cleaning and through the influenza epidemic, during which fifty-three members of the nursing staff were incapacitated.—Ed. T.H.]

